# Inhibition of the JAK and MEK Pathways Limits Mitochondrial ROS Production in Human Saphenous Vein Smooth Muscle Cells

**DOI:** 10.3390/cells15020159

**Published:** 2026-01-15

**Authors:** Israel O. Bolanle, James P. Hobkirk, Mahmoud Loubani, Roger G. Sturmey, Timothy M. Palmer

**Affiliations:** 1 Biomedical Institute for Multimorbidity, Centre for Biomedicine, Hull York Medical School, University of Hull, Hull HU6 7RX, UK; 2 School of Cardiovascular & Metabolic Health, British Heart Foundation Glasgow Cardiovascular Research Centre, University of Glasgow, Glasgow G12 8TA, UK; james.hobkirk3@nhs.net; 3 Department of Cardiothoracic Surgery, Castle Hill Hospital, Cottingham HU16 5JQ, UK; mahmoud.loubani@nhs.net

**Keywords:** reactive oxygen species, JAK/STAT, MAPK/ERK1,2, Type 2 diabetes mellitus

## Abstract

**Highlights:**

**What are the main findings?**

- Ruxolitinib and trametinib, JAK and MEK inhibitors, respectively, limit mitochondrial-derived ROS (mROS) production in human saphenous vein smooth muscle cells (HSVSMC), which is responsible for the maladaptive remodelling leading to saphenous VGF.
- Basal mROS level is higher in HSVSMCs from type 2 diabetic patients (T2DM) compared with non-diabetic patients.

**What are the implications of the main findings?**

- The JAK and MEK pathways are potential targets to reduce T2DM-dependent increased mROS production.
- The JAK and MEK pathways present viable targets for drug development to limit ROS-driven VGF.

**Abstract:**

Activation of JAK/STAT and MAPK/ERK1,2 signalling pathways has been shown to increase the production of reactive oxygen species (ROS) in multiple cell types involved in cardiovascular diseases (CVDs), including vascular smooth muscle cells (VSMCs). However, these have not yet been studied in human saphenous vein SMCs (HSVSMCs) responsible for the maladaptive remodelling leading to saphenous vein graft failure (VGF), to which patients with type 2 diabetes mellitus (T2DM) are more susceptible. Therefore, this study aimed to evaluate the contributions of the JAK/STAT and MAPK/ERK1,2 pathways towards production of mitochondrial ROS (mROS) in HSVSMCs from T2DM patients versus non-diabetic controls. HSVSMCs explanted from surplus HSV tissues from consenting patients undergoing coronary artery bypass graft surgery were stimulated in vitro with mitogenic stimuli known to be involved in neointimal hyperplasia (NIH) and VGF, which are known activators of the JAK/STAT and the MAPK/ERK1,2 signalling pathways. Flow cytometry was then used to analyse the production of mROS (superoxide) in MitoSOX-stained HSVSMCs. Additionally, we examined the effect of ruxolitinib and trametinib, selective inhibitors of JAK1/2 and MEK1/2 signalling pathways, respectively, on mROS levels in these cells. From our findings, mROS production was significantly higher in HSVSMCs from T2DM patients versus non-diabetic controls. Activation of either the JAK/STAT or MAPK/ERK1,2 signalling pathways did not significantly alter the production of mROS in HSVSMCs from both T2DM and non-diabetic patients. However, inhibition of JAK/STAT and MAPK/ERK1,2 signalling pathways with ruxolitinib and trametinib, respectively, resulted in a significant reduction in mROS in HSVSMCs from both T2DM and non-diabetic patients. Our findings demonstrate a JAK/STAT- and MAPK/ERK1,2-mediated production of mROS in HSVSMCs. Hence, they are potential targets for drug development to limit ROS production in ROS-driven proliferation and migration of HSVSMCs responsible for VGF.

## 1. Introduction

Cardiometabolic disorders such as type 2 diabetes mellitus (T2DM) have been linked to oxidative stress, which is an imbalance between reactive oxygen species (ROS) production and antioxidant buffering capacity [1]. At physiological levels, ROS act as signalling mediators and control many physiological processes, including the growth, proliferation, and migration of ECs and VSMCs, the formation and growth of new blood vessels, vascular tone, host defence, and genomic stability [1]. However, at pathophysiological levels, ROS alter cellular redox status and can contribute to the onset of cardiometabolic disorders such as T2DM, a major risk factor for several cardiovascular diseases (CVDs) [1,2]. Additionally, a large body of literature has described how increased ROS activity promotes maladaptive vascular cell proliferation and vascular dysfunction responsible for vein graft failure (VGF) [3,4].

ROS affect several signalling pathways such as the JAK/STAT and MAPK/ERK1,2 pathways, but the mechanisms by which ROS interact with cell-signalling proteins, how those proteins affect the level of intracellular ROS in turn, and the molecular interactions between distinct ROS-associated signalling pathways are not fully understood [5,6]. It has been suggested that activation of STAT family transcription factors is modulated by ROS signalling [7,8]. For instance, acute exposure of fibroblasts and A431 carcinoma cells to H_2_O_2_ is sufficient to activate STAT1 and STAT3 within 5 min [9]. This suggests that ROS are important intracellular messengers that control STAT activation [9]. On the other hand, the suppression of ROS production by antioxidants prevents ERK1,2 activation following stimulation with cellular stimuli [10,11], demonstrating the involvement of ROS in the activation of ERK1,2 pathways [11]. Oxidative modification of signalling proteins by ROS may be one possible mechanism for the activation of the MAPK/ERK1,2 pathway, since ROS can react with key amino acid residues to alter protein structure and function [12]. For example, ROS inhibits MAPK phosphatases (MKPs) such as dual-specific protein phosphatase-6 and MKP-8, enzymes responsible for dephosphorylating and deactivating the MAPK pathway, by oxidising conserved active site cysteine residues [13].

In numerous cell types, including vascular smooth muscle cells (VSMCs) and endothelial cells (ECs) linked to CVDs, activators of JAK/STAT and MAPK/ERK1,2 signalling pathways, such as interleukin 6 (IL-6), platelet-derived growth factor (PDGF), angiotensin II (Ang II) and thrombin, have been shown to stimulate ROS generation [14,15,16,17]. However, these have not yet been assessed in human saphenous vein SMCs (HSVSMCs) despite their significance in the aetiology of neointimal hyperplasia (NIH) responsible for long-term saphenous vein graft failure (VGF) in coronary artery bypass graft procedures [3]. Hence, our aim in this study was to investigate the effect of the activation and inhibition of the JAK/STAT and MAPK/ERK1,2 signalling pathways on the generation of mitochondrial-derived ROS (mROS) (O_2_^·^) production by HSVSMCs from non-diabetic and T2DM patients. We focused on the mitochondria because they are a principal source of cellular ROS and major mediators of cellular redox homeostasis due to their role in ROS production and dissipation [18,19].

## 2. Materials and Methods

### 2.1. Materials

All materials used in this study are listed in the Supplementary Information.

### 2.2. Isolation and Characterisation of HSVSMCs

As shown in Table 1, surplus HSV tissues were obtained from consenting T2DM and non-diabetic patients undergoing CABG surgery at the Hull University Teaching Hospitals NHS Trust Department of Cardiothoracic Surgery, Castle Hill Hospital, Cottingham, UK, under UK Health Research Authority ethical approval (NHS REC:15/NE/0138). The detailed procedures used to isolate, characterise, and culture primary HSVSMCs have been extensively described previously [20].

### 2.3. Preparation of Cell Samples for Flow Cytometry

HSVSMCs from non-diabetic and T2DM patients were cultured in 10 mm dish until 70–90% confluent. Following this, cells were treated with 0.1 μM ruxolitinib for 90 min, and then 1:1000 IL-6 (5 ng/mL) and sIL-6Rα (25 ng/mL) (IL-6/sIL-6Rα) or 10 ng/L PDGF-BB was used to treat the cells over the course of 24 h. Also, cells were treated with 10 nM trametinib for 90 min followed by treatment with 100 nM Ang II or 1 U/mL thrombin over the course of 24 h. All the concentrations of drugs used above were predetermined to be effective in HSVSMCs [21]. While 0.1 μM ruxolitinib significantly inhibited IL-6/sIL-6Rα-mediated phosphorylation of STAT3 on Tyr705, on the other hand, 10 nM trametinib inhibited the activation of ERK1,2 in HSVSMCs. Also, the activators of the pathways were confirmed to be effective at the used concentrations [21].

On the day of assay, untreated cells (control) and treated HSVSMCs were transferred into 1.5 mL centrifuge tubes and spun at 654× *g* (~1000 rpm) at 4 °C for 5 min. Pelleted cells were washed thrice with 1 mL of 1% (*w*/*v*) BSA in PBS. Using a haemocytometer, 5 × 10^5^ cells were resuspended in 100 µL 1% (*w*/*v*) BSA in PBS. Resuspended cells were incubated with 5 μM MitoSOX for 20 min at 37 °C, in a CO_2_ incubator. After this, cells were pelleted at 500× *g* for 5 min at 4 °C, and the supernatant was discarded. Pelleted cells were washed twice with 1% (*w*/*v*) BSA in PBS at 500× *g* for 5 min at 4 °C. Samples were kept on ice until flow cytometry analysis.

### 2.4. FACS Analysis with Fortessa Flow Cytometer

**Figure 1:**
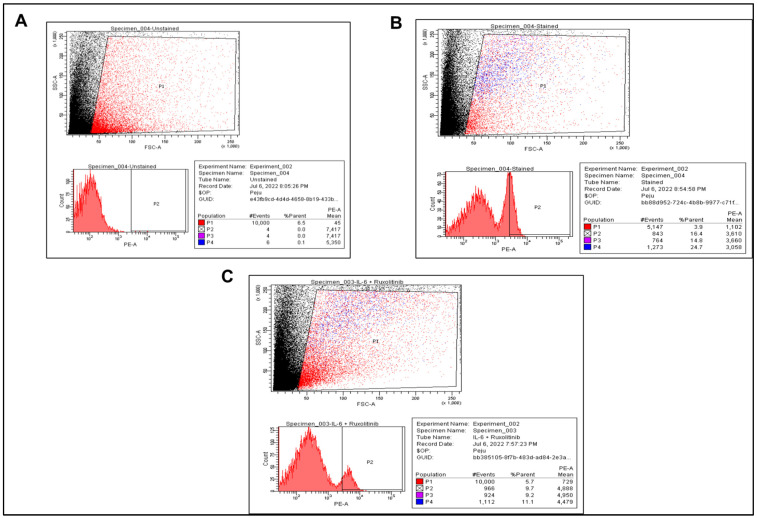
Gating strategy used to quantify the level of mROS production in HSVSMCs following MitoSOX staining. As already described in Section 2.2, prepared cells were incubated with 5 μM MitoSOX for 20 min at 37 °C, in a cell culture incubator. After this, cells were pelleted at 500× *g* for 5 min at 4 °C, and the supernatant was discarded. Pelleted cells were washed twice with 1% (*w*/*v*) BSA in PBS at 500× *g* for 5 min at 4 °C. Samples were kept on ice until flow cytometry analysis. A window with FSC as the horizontal axis and SSC as the vertical axis was created (top panel of (**A**–**C**)), and a loose gate that excludes debris found at the left corner of the FSC/SSC plots was created. This gate, labelled P1, helps to exclude dead cells from the preparation process and debris from the assay. Another gate (P2—bottom left panel of (**A**–**C**)) that included MitoSOX-positive cells (blue stains in the cell population shown in the top panel of (**A**–**C**)) but excluded MitoSOX-negative cells (red stains in the cell population shown in the top panel of (**A**–**C**)) was drawn from the P1 population (top panel of (**A**–**C**)). The percentage of cells within P1 that shifted into P2 was determined and acquired as %PE-A (bottom right table of (**A**–**C**)). (**A**) Unstained control, (**B**) Untreated cells with MitoSOX stain, and (**C**) Treated cells with MitoSOX stain are shown with representative dot plots and data summary of the different treatment conditions and gating strategies.

The expression of MitoSOX, used as a marker for mROS production, was quantified in a calibrated BD LSRFortessa™ Cell Analyzer (BD Bioscience) via the PE channel [22]. The acquired data were processed using Diva Software (Version 8). To reproduce the ideal PMT voltages for every panel, the acquired data were standardised using CS&T beads and SPHERO™ Rainbow Profile Calibration Particles. Compensated data were exported for analysis in FlowJO™ (BD) and gated as shown (Figure 1). In brief, a window with FSC as the horizontal axis and SSC as the vertical axis was created, and a loose gate that excludes debris found at the left corner of the FSC/SSC plots was created. This gate, labelled P1, helps to exclude dead cells from the preparation process and debris from the assay. Another gate (P2) that included MitoSOX-positive cells but excluded MitoSOX-negative cells was drawn from the P1 population. The percentage of cells within P1 that shifted into P2 was determined and acquired as %PE-A. %PE-A is the percentage of the viable cells that responded to MitoSOX stain, which is a readout of mROS production. This procedure was adopted for all treatment conditions. Six (*n* = 6) biological replicates were used for statistical analysis.

### 2.5. Statistical Analysis

Statistical analysis was carried out using GraphPad Prism^®^ 6, San Diego, CA, USA. Data are presented as mean ± standard error of mean (S.E.M) and were analysed using an independent *t*-test and both one-way and two-way analysis of variance, followed by both Dunnett’s and Holm–Šidák’s post hoc tests to determine significant differences between means where *p* < 0.05 was considered statistically significant.

## 3. Results

### 3.1. Effects of JAK Inhibition on mROS Production in HSVSMCs from Non-Diabetic and T2DM Patients

**Figure 2:**
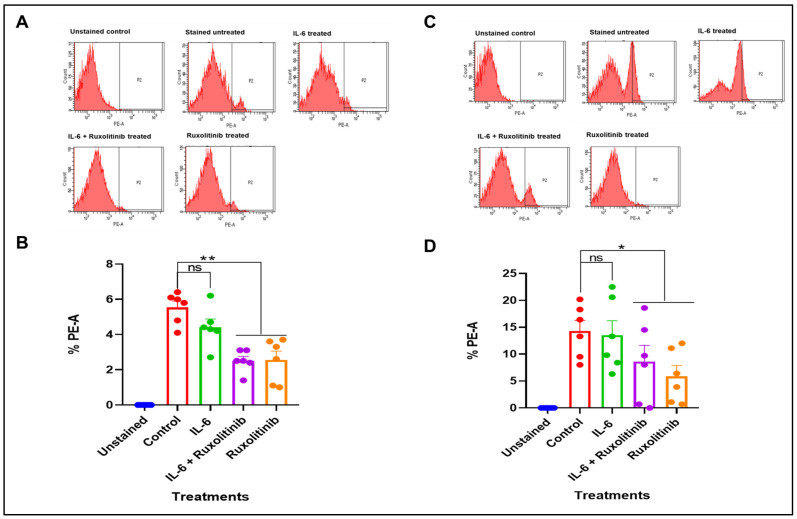
Flow cytometry analysis of mROS production in HSVSMCs from non-diabetic and T2DM patients after treatment with IL-6 (5 ng/mL) and sIL-6Rα (25 ng/mL) (IL-6/sIL-6Rα) +/− 0.1 μM ruxolitinib. (**A**) Flow cytometry chart showing the percentage of MitoSOX-positive HSVSMCs from non-diabetic patients after labelled treatments. The cell population that shifts to the right represents cells that are positive to the MitoSOX stain, which correlates to the generation of mROS. (**B**) Comparison of the percentage of HSVSMCs from non-diabetic patients that are positive to the MitoSOX staining after treatment with 1:1000 IL-6 (5 ng/mL) and sIL-6Rα (25 ng/mL) (IL-6/sIL-6Rα) +/− 0.1 μM ruxolitinib. (**C**) Flow cytometry chart showing the percentage of MitoSOX-positive HSVSMCs from T2DM patients after labelled treatments. The cell population that shifts to the right represents cells that are positive to the MitoSOX stain, which correlates to the generation of mROS. (**D**) Comparison of the percentage of HSVSMCs from T2DM patients that are positive to the MitoSOX staining after treatment with 1:1000 IL-6 (5 ng/mL) and sIL-6Rα (25 ng/mL) (IL-6/sIL-6Rα) +/− 0.1 μM ruxolitinib. Data are presented as mean ± SEM from *n* = 6 biological replicates using HSVSMC samples from different non-diabetic and T2DM patients as appropriate, * *p* < 0.05, ** *p* < 0.01, ns—non-significant. All data were compared to the untreated control cells for statistical significance. IL-6: IL-6/sIL-6Rα.

While mitochondrial ROS production has been shown to increase in cardiac cells in response to activation of STAT3 by cytokines like IL-6 and growth factors like PDGF [23,24], it is currently unclear whether the JAK/STAT pathway impacts mROS production in VSMCs such as those from HSVs. However, these activators can concurrently increase NADPH oxidase activity [23,24]. In addition, numerous studies have established NADPH oxidase as the main source of ROS in human failing myocardium and cardiac remodelling, primarily through actions on redox-sensitive signal transduction pathways [25,26,27,28,29]. It is unclear to what extent the JAK/STAT pathway contributes to the total amount of ROS produced and of what significance this is in driving cardiac pathologies. Most importantly, no study has examined this in HSVSMCs, despite its role in the aetiology of NIH. Therefore, we investigated the role of the JAK/STAT pathway on the generation of mROS in HSVSMCs from both non-diabetic and T2DM patients. To assess this, we used JAK1/2-selective inhibitor ruxolitinib to assess the sensitivity of mROS production in HSVSMCs from non-diabetic and T2DM patients in response to inhibition of the JAK/STAT pathway. As shown in Figure 2A, IL-6/sIL-6Rα stimulation did not result in any significant alteration in the proportion of cells that respond to MitoSOX stain, which is used in this instance to measure mROS levels in HSVSMCs from non-diabetic patients. %PE-A in stained and unstimulated HSVSMCs from T2DM patients was used as the control, and there was no significant difference when compared with %PE-A from IL-6/sIL-6Rα-stimulated cells. However, at a concentration (0.1 mM) which we have previously shown to be effective in blocking JAK/STAT signalling in HSVMCs [21], ruxolitinib caused a significant reduction versus untreated control cells (*p* < 0.01, *n* = 6), denoting a significant decrease in mROS levels (Figure 2B). On the other hand, IL-6/sIL-6Rα did not significantly alter mROS generation in HSVSMSCs from T2DM patients (Figure 2C). However, similar to non-diabetic patients, ruxolitinib significantly decreased mROS levels in HSVSMCs from T2DM patients with or without IL-6/sIL-6Rα versus untreated control cells (*p* < 0.05, *n* = 6; Figure 2D).

#### 3.1.1. Effect of PDGF-BB and Ruxolitinib on mROS Production in HSVSMCs from Non-Diabetic and T2DM Patients

**Figure 3:**
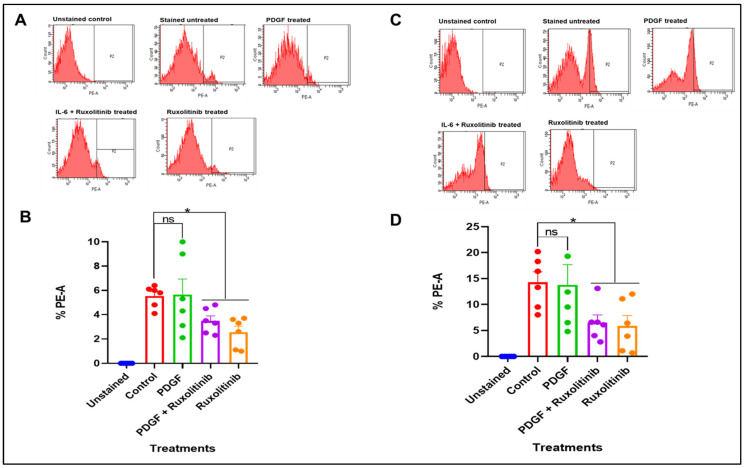
Flow cytometry analysis of mROS production in HSVSMCs from non-diabetic and T2DM patients after treatment with 10 ng/L PDGF-BB +/− 0.1 μM ruxolitinib. (**A**) Flow cytometry chart showing percentage of MitoSOX-positive HSVSMCs from non-diabetic patients after labelled treatments. The cell population that shifts to the right represents cells that are positive to the MitoSOX stain, which correlates to the generation of mROS. (**B**) Comparison of the percentage of HSVSMCs from non-diabetic patients that are positive to MitoSOX staining after treatment with 10 ng/L PDGF-BB +/− 0.1 μM ruxolitinib. (**C**) Flow cytometry chart showing percentage of MitoSOX-positive HSVSMCs from T2DM patients after labelled treatments. The cell population that shifts to the right represents cells that are positive to the MitoSOX stain, which correlates to the generation of mROS. (**D**) Comparison of the percentage of HSVSMCs from T2DM patients that are positive to the MitoSOX staining after treatment with 10 ng/L PDGF-BB +/− 0.1 μM ruxolitinib. Data are presented as mean ± SEM from *n* = 6 biological replicates using HSVSMC samples from different non-diabetic and T2DM patients as appropriate, * *p*< 0.05, ns—non-significant. All data were compared to untreated control cells for statistical significance. PDGF: PDGF-BB.

Figure 3A shows the production of mROS, as determined by %PE-A in HSVSMCs from non-diabetic patients when treated with PDGF-BB +/− ruxolitinib and ruxolitinib compared with untreated control cells. The control was the %PE-A in unstimulated stained HSVSMCs from non-diabetic patients. Our results showed that there was no significant alteration in mROS level in HSVSMCs from non-diabetic patients in response to PDGF-BB treatment for 24 h. However, ruxolitinib alone caused a significant reduction in mROS level (*p* < 0.05, *n* = 6 versus untreated control cells; Figure 3B).

On the other hand, Figure 3C shows there was also no significant alteration in mROS level after stimulation of HSVSMCs from T2DM patients with PDGF-BB versus untreated control cells (Figure 3C). However, there was a significant reduction in mROS production in HSVSMCs from T2DM patients when treated with ruxolitinib alone (*p* < 0.05, *n* = 6 versus untreated control cells; Figure 3D).

#### 3.1.2. Comparing the Level of mROS in HSVSMCs from Non-Diabetic and T2DM Patients at Basal and After Treatment with IL-6/sIL-6Rα +/− Ruxolitinib and PDGF +/− Ruxolitinib

**Figure 4:**
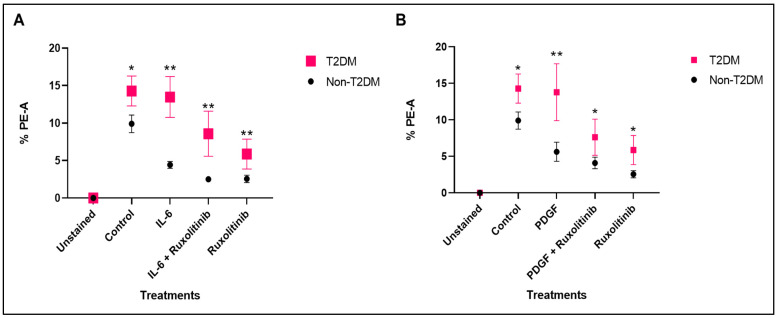
Comparison of the mROS production in HSVSMCs from non-diabetic and T2DM patients after treatment with IL-6 (5 ng/mL) and sIL-6Rα (25 ng/mL) (IL-6/sIL-6Rα) +/− 0.1 μM ruxolitinib and 10 ng/L PDGF-BB +/− 0.1 μM ruxolitinib. (**A**) Comparison of mROS generated by HSVSMCs from non-diabetic and T2DM patients after treatment with IL-6/sIL-6Rα +/− ruxolitinib. (**B**) Comparison of mROS generated by HSVSMCs from non-diabetic and T2DM patients after treatment with PDGF +/− ruxolitinib. Data are presented as mean ± SEM from *n* = 12 for untreated control cells and *n* = 6 for treated samples of biological replicates using HSVSMC samples from different patients, * *p* < 0.05, ** *p*< 0.01. IL-6: IL-6/sIL-6Rα, PDGF: PDGF-BB.

The production of mROS in HSVSMCs from T2DM and non-diabetic patients with or without IL-6/sIL-6Rα stimulation with or without ruxolitinib was also directly compared. Interestingly, this showed that unstimulated HSVSMCs from T2DM patients produced significantly more mROS than those from non-diabetic patients (Figure 4A; *p* < 0.05, *n* = 12). Also, mROS level in HSVSMCs from T2DM patients was consistently higher (Figure 4A; *p* < 0.01, *n* = 6) after treatments with IL-6/sIL-6Rα, IL-6/sIL-6Rα + ruxolitinib, and ruxolitinib alone when compared with those from non-diabetic control HSVSMCs.

Similarly, a direct comparison of the mROS levels in HSVSMCs from T2DM and non-diabetic patients with or without PDGF-BB stimulation ± ruxolitinib showed that in unstimulated conditions, HSVSMCs from patients with T2DM produced significantly higher mROS than those from non-diabetic patients (Figure 4B; *p* < 0.05, *n* = 12). Also, the level of mROS in HSVSMCs from T2DM patients was also higher after treatment with PDGF-BB (Figure 4B; *p* < 0.01, *n* = 6). Additionally, when comparing the mROS levels following treatment with PDGF-BB + ruxolitinib versus ruxolitinib alone, the data showed that HSVSMCs from T2DM patients produced a significantly higher amount of mROS (Figure 4B; *p* < 0.05, *n* = 6).

### 3.2. Effects of MEK Inhibition on mROS Production in HSVSMCs from Non-Diabetic and T2DM Patients

The regulation of the MAPK/ERK1,2 pathway by ROS has garnered a lot of interest as the MAPK/ERK1,2 pathway mediates both mitogen- and stress-activated signals, and ROS have important functions such as signalling molecules and controllers of cellular activity [6]. Increasing evidence suggests that ROS play a physiological role as “second messengers” in processes controlling cell growth, proliferation, migration, and death [12]. Although the exact mechanisms are still unclear, several studies have described the activation of the MAPK/ERK1,2 pathway by ROS and the functional consequences in the pathogenesis of cardiovascular disorders such as ischemia, cardiac hypertrophy, cardiac remodelling after myocardial infarction, atherosclerosis, and vascular restenosis [6,10,12,30]. Despite these advances, the impact that the MAPK/ERK1,2 pathway has on the generation of ROS in SMCs is not well known. Hence, we examined the effect of pre-treatment with MEK1/2-selective inhibitor, trametinib, on mROS generation in response to Ang II and thrombin, two stimuli that activate the MAPK/ERK1,2 pathway in HSVSMCs [21]. To achieve this, we measured mROS levels following treatment with Ang II/thrombin with/without trametinib, and trametinib alone. We also assessed whether T2DM status impacts ROS generation under the various treatment conditions versus non-diabetic controls.

#### 3.2.1. Effect of Ang II and Trametinib on mROS Production in HSVSMCs from Non-Diabetic and T2DM Patients

**Figure 5:**
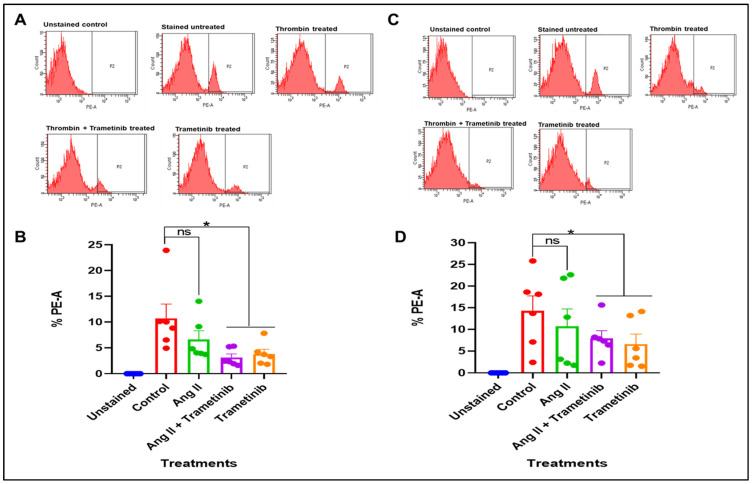
mROS production in HSVSMCs from non-diabetic and T2DM patients after treatment with 100 nM Ang II +/− 10 nM trametinib. (**A**) Flow cytometry chart showing the percentage of MitoSOX-positive HSVSMCs from non-diabetic patients after labelled treatments. The cell population that shifts to the right represents cells that are positive to the MitoSOX stain, which correlates to the generation of mROS. (**B**) Comparison of the percentage of HSVSMCs from non-diabetic patients that are positive to the MitoSOX staining after treatment with 100 nM Ang II +/− 10 nM trametinib. (**C**) Flow cytometry chart showing the percentage of MitoSOX-positive HSVSMCs from T2DM patients after labelled treatments. The cell population that shifts to the right represents cells that are positive to the MitoSOX stain, which correlates to the generation of mROS. (**D**) Comparison of the percentage of HSVSMCs from T2DM patients that are positive to the MitoSOX staining after treatment with 100 nM Ang II +/− 10 nM trametinib. Data are presented as mean ± SEM from *n* = 6 biological replicates using HSVSMC samples from different non-diabetic and T2DM patients as appropriate, * *p* < 0.05, ns—non-significant. All data were compared to the untreated control cells for statistical significance. Ang II: Angiotensin II.

We examined mROS level in HSVSMCs from non-diabetic patients at the basal level and following treatment with Ang II with or without trametinib versus untreated cells. There was no significant alteration in mROS level in HSVSMCs from non-diabetic patients after stimulation with Ang II when compared with untreated cells (Figure 5A). However, at a concentration (10 nM) that we have previously shown to significantly inhibit MAPK/ERK1,2 phosphorylation in HSVSMCs [21], trametinib caused a significant reduction in mROS levels with or without Ang II (Figure 5B; *p* < 0.05, *n* = 6 versus untreated control cells). Additionally, as shown in Figure 5C, there was no significant alteration in the level of mROS after stimulating HSVSMCs from T2DM patients with Ang II compared to untreated controls. However, trametinib also caused a significant reduction in mROS level in HSVSMCs with or without treatment with Ang II (Figure 5D; *p* < 0.05, *n* = 6 versus untreated control cells).

#### 3.2.2. Effect of Thrombin and Trametinib on mROS Production in HSVSMCs from Non-Diabetic and T2DM Patients

**Figure 6:**
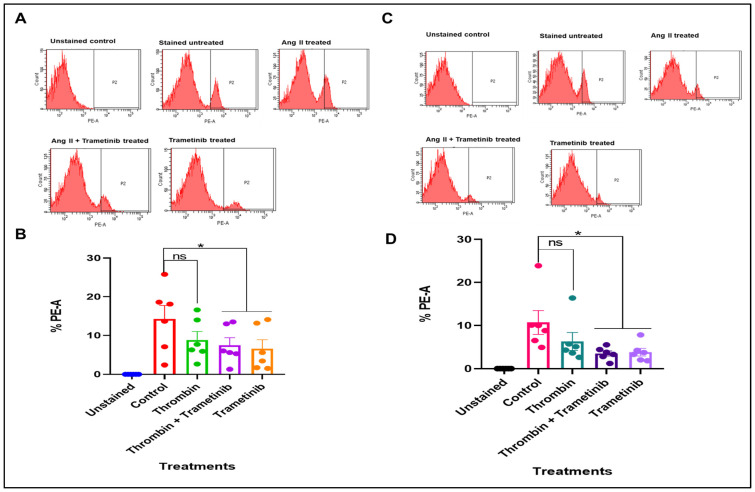
Flow cytometry analysis of mROS production in HSVSMCs from non-diabetic and T2DM patients after treatment with 1 U/mL thrombin +/− 10 nM trametinib. (**A**) Flow cytometry chart showing the percentage of MitoSOX-positive HSVSMCs from non-diabetic patients after labelled treatments. The cell population that shifts to the right represents cells that are positive to the MitoSOX stain, which correlates to the generation of mROS. (**B**) Comparison of the percentage of HSVSMCs from non-diabetic patients that are positive to the MitoSOX staining after treatment with 1 U/mL thrombin +/− 10 nM trametinib. (**C**) Flow cytometry chart showing the percentage of MitoSOX-positive HSVSMCs from T2DM patients after labelled treatments. The cell population that shifts to the right represents cells that are positive to the MitoSOX stain, which correlates to the generation of mROS. (**D**) Comparison of the percentage of HSVSMCs from T2DM patients that are positive to the MitoSOX staining after treatment with 1 U/mL thrombin +/− 10 nM trametinib. Data are presented as mean ± SEM from *n* = 6 biological replicates using HSVSMC samples from different non-diabetic and T2DM patients as appropriate, * *p* < 0.05, ns—non-significant. All data were compared to the untreated control cells for statistical significance.

Figure 6A shows the production of mROS in HSVSMCs from non-diabetic patients when treated with thrombin with or without trametinib and trametinib alone compared with untreated control cells. As shown, there was no significant alteration in mROS level in HSVSMCs from non-diabetic patients after stimulation with thrombin when compared with untreated control cells. Trametinib, however, caused a significant reduction (Figure 6B, *p* < 0.05, *n* = 6 compared with unstimulated control cells) in mROS generation with or without thrombin. Furthermore, Figure 6C shows that there was no significant alteration in the production of mROS after stimulation of HSVSMCs from T2DM patients with thrombin compared with the untreated control cells. However, trametinib caused a significant reduction (Figure 6D, *p* < 0.05, *n* = 6 compared with untreated control cells) in ROS production in HSVSMCs from T2DM patients with or without thrombin.

#### 3.2.3. Comparing the Level of mROS in HSVSMCs from Non-Diabetic and T2DM Patients at Basal and After Treatment with Ang II and Thrombin +/− Trametinib

**Figure 7:**
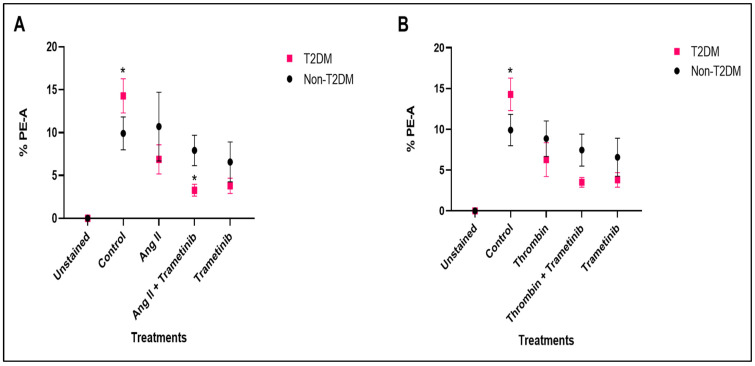
Comparison of the mROS production in HSVSMCs from non-diabetic and T2DM patients after treatment with 100 nM Ang II +/− 10 nM trametinib and 1 U/mL thrombin +/− 10 nM trametinib. (**A**) Comparison of mROS generated by HSVSMCs from non-diabetic and T2DM patients after treatment with Ang II +/− trametinib. (**B**) Comparison of mROS generated by HSVSMCs from non-diabetic and T2DM patients after treatment with thrombin +/− trametinib. Data are presented as mean ± SEM from *n* = 12 for untreated control cells and *n* = 6 for treated samples of biological replicates using HSVSMC samples from different patients, * *p*< 0.05. Ang II: Angiotensin II.

We then directly compared mROS levels in HSVSMCs from T2DM and non-diabetic patients treated with or without Ang II ± trametinib. The results showed that untreated HSVSMCs from T2DM patients produced significantly higher mROS than those from untreated non-diabetic controls (Figure 7A; *p* < 0.05, *n* = 6). In contrast, there was no significant difference in mROS levels after treatment with either Ang II or trametinib. However, comparing mROS levels following treatment with Ang II + trametinib, there was a significant reduction in mROS generated in T2DM (Figure 7A; *p* < 0.05, *n* = 6). Also, the mROS levels in HSVSMCs from T2DM and non-diabetic patients with or without thrombin stimulation with or without trametinib treatment were directly compared in Figure 7B. The results showed that untreated HSVSMCs from patients with T2DM produced significantly higher (*p* < 0.05, *n* = 6) mROS than those from non-diabetic controls. However, there was no significant difference in mROS production after treatment with either thrombin, thrombin + trametinib, or trametinib alone.

## 4. Discussion

In this study, we have evaluated the contributions of the JAK/STAT and MAPK/ERK1,2 signalling pathways on mROS generation in HSVSMCs from T2DM and non-diabetic patients. In numerous cell types, including VSMCs, ECs, and platelets, which have all been linked to CVDs, activators of these signalling pathways, such as IL-6, PDGF, Ang II, and thrombin, have been shown to stimulate ROS production [14,15,16,17,31]. However, these have not yet been assessed in HSVSMCs despite their significance in the aetiology of NIH and VGF. For the first time, we have now evaluated this using flow cytometry to analyse ROS production by staining cells with MitoSOX, a tool widely used for live-cell imaging to measure mitochondria superoxide production [22,32,33]. MitoSOX penetrates live cells to target mitochondria and is rapidly and selectively oxidised by superoxide but not other ROS. As the oxidised products are highly fluorescent, they can be readily detected by flow cytometry [22,32,33].

From our findings, IL-6/sIL-6Rα did not cause any significant alteration in the production of mROS in HSVSMCs from both non-diabetic and T2DM patients (Figure 2). This is the first time this experiment is described in HSVSMCs from T2DM and non-diabetic patients, and it is not entirely clear why IL-6/sIL-6Rα did not enhance the generation of mROS in the samples. However, it has been previously shown in a mouse model of early diabetic retinopathy that inhibiting IL-6 *trans*-signalling with recombinant sgp130Fc reduces oxidative stress caused by an increase in ROS production [34]. Also, there exists a substantial body of evidence connecting IL-6 to the onset and progression of CVDs through changes to vascular function, decreased NO levels, and increased vascular superoxide levels [35,36,37,38,39,40]. More so, increases in vascular superoxide may play a key role in the relationship between endothelial dysfunction and the activation of inflammatory molecules and pathways such as JAK/STAT signalling in CVDs [41,42,43]. Emerging evidence reveals that inflammatory cytokines, such as IL-6, influence the production and activity of both eNOS and NADPH oxidase, affecting the amounts of NO and superoxide which contribute to oxidative stress [41,42,43,44]. Therefore, together with our findings, these observations suggest that the effect of IL-6 on ROS/oxidative stress is via these pathways, which are present in the plasma membrane and intracellular membranes, rather than mROS generation.

Furthermore, neither HSVSMCs from non-diabetic (Figure 3A) nor those from T2DM patients (Figure 3C) showed significant alterations in the levels of mROS generation after treatment with PDGF-BB. Numerous studies have shown that PDGF-BB induces the production of ROS in a variety of cell types, including ECs, human lens epithelial cells, renal tubular cells, and adipocytes [45,46,47,48,49], but these studies have only shown that PDGF-BB achieves this via activation of NADPH oxidases. Additionally, it has been demonstrated that chicoric acid, a purified isolate from plants and vegetables, attenuated a PDGF-BB-induced VSMC proliferation and migration via inhibition of ROS accumulation [50]. Also, it has been suggested that PDGF-BB contributes to the production of ROS in SMCs [45], although the exact mechanism (s) involved are not fully understood. Therefore, our observation further suggests that despite the activation of the JAK/STAT signalling pathway, there might be a limited contribution to mROS generation in HSVSMCs.

While there were no significant alterations in mROS production in HSVSMCs from both T2DM and non-diabetic patients after treatment with IL-6/sIL-6R and PDGF-BB, inhibition of JAK1/2 with ruxolitinib caused a significant reduction in the production of mROS in HSVSMCs from both T2DM and non-diabetic controls (Figure 2 and Figure 3). Chronic inflammation and oxidative stress typically co-exist in a pathological intracellular environment and are closely associated processes [51]. It is widely acknowledged that inflammatory cells serve as a source of ROS, which are, in turn, able to amplify the activation of pro-inflammatory signalling pathways. However, the mechanism behind the pathophysiologic interaction between ROS production and the inflammatory response is not well understood [51]. Our findings provide new insight into how JAK inhibition might be used to decrease ROS generation. The fact that this is the first description in HSVSMCs, a crucial cell type involved in the vascular dysfunction that results in saphenous VGF seen in T2DM patients, is significant. Interestingly, JAK inhibitors have been suggested as potential therapies to control oxidative stress [52,53]. For instance, the oxidative stress response is upregulated in activated salivary gland epithelial cells from patients with Primary Sjögren’s syndrome [52]. More so, it has been demonstrated that JAK inhibitors, AG490 and ruxolitinib, can both reverse ICAM-1- and PD-L1-mediated activation of salivary gland epithelial cells brought on by ROS by activating STAT3 [52].

On the other hand, our findings showed that HSVSMCs from neither non-diabetic T2DM (Figure 5A) nor T2DM patients (Figure 5C) had any significant changes in the amounts of mROS generation following stimulation of the MAPK/ERK1,2 pathway with Ang II. Through the generation of ROS, Ang II is known to promote VSMC growth, hypertrophy, and/or hyperplasia, and inflammation, which promote the development of hypertension, atherosclerosis, heart failure, and restenosis after vascular injury [54,55,56,57,58,59,60,61,62]. Superoxide and hydrogen peroxide are produced when Ang II activates NADPH oxidase. These two mediators may then interact with intracellular growth-related proteins and enzymes such as p38, ERK1,2, and AKT/PKB to mediate the physiological responses, such as VSMC proliferation [62]. Additionally, in vivo stimulation with Ang II does not result in hypertension in mice lacking the cytosolic NADPH oxidase subunit p47^phox^, a mouse model with NADPH oxidase defects [63]. According to this study, Ang II-induced NADPH oxidase activation is responsible for the vascular ROS produced in the endothelium, adventitia, and VSMCs that are linked to hypertension [63].

The mechanisms through which ROS control cell-signalling proteins, and how they affect the amount of intracellular ROS in turn, and the intricate relationships between diverse ROS-linked signalling pathways are unclear and complex [5]. Studies have demonstrated how ROS influences the activation of several signalling pathways such as the NF-*κ*B, Keap1-Nrf2-ARE, MAPKs/ERK, and PI3K-Akt pathways [5,64,65,66,67]. However, little is known about how the activation of these pathways influences the generation of ROS. Our findings have now given further insights with regard to activation of the MAPK/ERK 1,2 pathway in HSVSMCs, and the results suggest that the production of mROS, specifically superoxide, is not altered.

Also, stimulation of the MAPK/ERK1,2 pathway with thrombin in HSVSMCs from either non-diabetic (Figure 6A) or T2DM patients (Figure 6C) did not alter the generation of mROS. This finding further supports the notion that activation of the MAPK/ERK1,2 signalling pathway had no impact on mROS generation in HSVSMCs from either non-diabetic or T2DM patients, similar to the results with Ang II. Although thrombin has been shown to promote the generation of ROS in platelets [17,68]. More so, ROS are known to play important roles in intra-platelet signalling and subsequent platelet activation. Although PAR1 and PAR4 are known as the major thrombin receptors on platelets, thrombin can communicate with human platelets via GPIb [69]. The receptors and signalling pathways involved in thrombin-induced ROS formation are still not completely understood. However, it has now been suggested that GPIb and PAR4 are both necessary for thrombin-induced ROS production in platelets, pointing to a novel functional partnership between the two proteins [17]. In addition to this vital contribution to knowledge, we have now demonstrated this relationship—the effect of thrombin-induced activation of the MAPK/ERK1,2 pathway and mROS production in HSVSMCs.

Furthermore, our findings show that inhibition of the MAPK/ERK1,2 downstream signalling pathway with MEK1/2 inhibitor, trametinib, significantly reduced mROS levels in HSVSMCs from both non-diabetic (Figure 6B) and T2DM patients (Figure 6D). Inhibiting ROS production has been shown to suppress the ERK1,2 and JNK signalling in the rat spinal cord following limb ischemia–reperfusion injury [70]. It was proposed that the activation of ERK1,2 and JNK in the spinal cord was mediated by the superoxide generated following hind limb ischemia and reperfusion [70]. This model supports earlier research showing how controlling ROS generation can be used to modulate the activation of the ERK1,2 pathway [5,66,70]. However, little is known about the reverse relationship. We have now demonstrated how inhibiting MAPK/ERK1,2 could be explored to reduce the production of mROS in HSVSMCs.

Another major finding from our study was that HSVSMCs from T2DM patients produced significantly more mROS than those from the non-diabetic controls, and the degree of mROS inhibition by ruxolitinib with or without IL-6/sIL-6Rα or PDGF-BB stimulation was similar in cells from T2DM patients and controls (Figure 4). This result is consistent with earlier studies showing increased ROS production in T2DM subjects compared to non-diabetic controls [71,72]. In addition, multiple studies, including the assessment of oxidative stress biomarkers in diabetic patients and T2DM rat models of diabetes, have suggested a clear connection between oxidative stress and diabetes [72,73]. Chronic hyperglycaemia-induced insulin resistance is also strongly suspected to be caused by oxidative damage [74]. Furthermore, production of mROS in HSVSMCs from T2DM was higher compared to those from non-diabetic patients (Figure 7). This is consistent with previously described findings (Figure 4), which showed that mROS production in HSVSMSC from T2DM was higher than that from the non-diabetic phenotype. However, the degree of inhibition by trametinib in T2DM subjects tends to be significantly higher (Figure 7) when compared with ruxolitinib (Figure 4).

Furthermore, while ruxolitinib caused a significant reduction in mROS level in ruxolitinib-treated HSVSMCs from T2DM patients compared with HSVSMCs from untreated T2DM controls (Figure 2 and Figure 3); however, it did not significantly reduce the level when compared with HSVSMCs from non-diabetic patients (Figure 4). On the other hand, trametinib not only reduced the mROS level in HSVSMCs from T2DM when compared with untreated T2DM controls (Figure 5 and Figure 6), but it also normalised the mROS level that there was no significant difference when compared with HSVSMCs from non-diabetic controls (Figure 7). The reason for these observations is currently not clearly known. Considering that increased ROS production promotes vascular restenosis, NIH, and eventual VGF capacity [1,3,4], our findings further give insight into a possible reason for the higher VGF observed in T2DM when compared with non-diabetic patients [3,75].

In summary, although our findings indicate that the JAK/STAT and MAPK/ERK1,2 signalling pathways are viable targets for reducing mROS production in HSVSMCs from both non-diabetic and T2DM patients, we acknowledge that more investigations will be required to further elucidate these initial findings. One important observation from our study was that, contrary to previous findings [14,15,16,17] where it has been demonstrated that the activators stimulate ROS generation in numerous cell types, including VSMSCs and ECs, these activators (IL-6, PDGF, Ang II, and thrombin) did not increase mROS production in HSVSMCs from both non-diabetic and T2DM patients, even though we have previously established that these concentrations significantly increased JAK1/2-mediated phosphorylation of STAT3 or ERK1,2 phosphorylation [21]. While the reason for this is currently unclear, it would be at best speculative to infer that these effects are cell-specific, as this is the first time that this experiment has been described in HSVSMCs. Also, our contrasting observation raises the question of whether mROS may have different kinetics from the assessed cytosolic signalling readouts. Therefore, additional assays such as assessing reduced mitochondrial electron transport chain activity, altered mitochondrial membrane potential, upregulation of antioxidant enzymes, and crosstalk with NADPH oxidase pathways, as well as determination of mitochondrial membrane potential, total cellular ROS, and mitochondrial mass would be useful to establish the JAK/STAT and MAPK/ERK1,2 signalling pathways as major targets to limit ROS-driven vascular dysfunction responsible for VGF. Additionally, as we have used HSVSMCs in this current study, repeating these experiments using intact saphenous vein tissue could offer additional strength to our conclusion.

## 5. Conclusions

From our findings, the activation of the JAK/STAT and MAPK/ERK1,2 signalling pathways did not appear to have significantly altered the production of mROS in HSVSMCs from non-diabetic and T2DM patients. However, inhibition of both signalling pathways, JAK/STAT and MAPK/ERK1,2, with ruxolitinib and trametinib, respectively, caused a significant reduction in the generation of mROS in HSVSMCs from both T2DM and non-diabetic patients. Importantly, our findings showed that mROS production in HSVSMSCs from T2DM was higher than that from non-diabetic phenotypes. Thus, these findings provide new insights and justification for further research aimed at identifying the mechanisms responsible and developing novel therapies that could improve vein graft patency in T2DM patients.

## Figures and Tables

**Table 1:** Patient information for HSV samples.

	Description	T2DM	Non-T2DM
1	Number of samples	17	38
2	Age	Range: 56–80; Mean: 69.2	Range: 47–84; Mean: 64.5
3	Sex	Male—15; Female—2	Male—36; Female—2
4	Ethnicity	White British—17	White British—37; Asian—1
5	Reason for surgery	CAD	CAD

CAD: coronary artery disease; T2DM: type 2 diabetes mellitus. HSV samples were collected between January 2020 and May 2022 from patients undergoing elective coronary artery bypass graft procedure (CABG).

## Data Availability

Data will be provided upon request.

## Supplementary Materials

The following supporting information can be downloaded at: https://www.mdpi.com/article/10.3390/cells15020159/s1, Supplementary Data.

## Abbreviations

The following abbreviations are used in this manuscript:

Ang II	angiotensin II
BSA	bovine serum albumin
CAD	coronary artery disease
CVD	cardiovascular disease
EC	endothelial cell
ERK	extracellular signal-regulated kinase
FSC	forward scatter
HSVSMC	human saphenous vein smooth muscle cell
IL-6	interleukin-6
JAK	janus kinase
MAPK	mitogen-activated protein kinase
mROS	mitochondrial-derived reactive oxygen species
NADPH	nicotinamide adenine dinucleotide phosphate
NF-Κb	nuclear factor kappa-light-chain-enhancer of activated B cells
NIH	neointimal hyperplasia
PBS	phosphate-buffered saline
PDGF	platelet-derived growth factor
ROS	reactive oxygen species
SSC	side scatter
STAT	signal transducer and activator of transcription
T2DM	type 2 diabetes mellitus
VGF	vein graft failure
VSMC	vascular smooth muscle cell
