# Supplementary material for: Inhibition of the JAK and MEK Pathways Limits Mitochondrial ROS Production in Human Saphenous Vein Smooth Muscle Cells
Source: Cells. 2026 Jan 15;15(2):159. doi: 10.3390/cells15020159 (PMC12839968; doi:10.3390/cells15020159)
Supplement: Supplementary file 1 [file cells-15-00159-s001.zip › cells-4055457-supplementary.pdf]

SUPPLEMENTARY DATA

1. Materials

Company/Supplier	Catalogue number
**Abcam, Cambridge, UK**	
Ruxolitinib	ab141356
**BD Bioscience, Berkshire, UK**	
BD facsflow™, Sheath Fluid	342003
FACS clean solution	340345
Detergent solution concentrate	660585
Facsuite[tm] CS&T Research beads	650621
BD facsflow™, Sheath Fluid	342003
**Lonza Ltd, Basal, Switzerland**	
Lonza DPBS 0.0095M (PO4) without Ca and Mg	LZBE17-512F
**PromoCell GmbH, Heidelberg, Germany**	
Smooth muscle cell growth medium 2 kit	C-22162
**R&D systems, Minnesota, USA**	
Recombinant human IL-6 protein	206-IL
Recombinant human IL-6 R alpha protein	227-SR-025

**Sarstedt, Nümbrecht, Germany**	
Tissue culture flasks, vented caps, 25 cm^2	83.3910.002
Tissue culture flasks, vented caps, 75 cm^2	83.3911.002
Serological Pipette 5ml Individually Wrapped Sterile Non-Pyrogenic	86.1253.001
**Sigma-Aldrich (Merck), Dorset, UK**	
Angiotensin II human	A9525-5mg
Thrombin from bovine plasma	T4648-1ku
**Stratech, Cambridgeshire, UK**	
Trametinib	GSK1120212
**Thermo Scientific, Massachusetts, USA**	
Mitosox red mitochondrial superoxide indicator 50µg	11579096
Recombinant human PDGF-BB	10531285
